# Association Between Peer Comparison Feedback and Hospitalist Antibiotic Prescribing

**DOI:** 10.1001/jamanetworkopen.2026.9504

**Published:** 2026-04-28

**Authors:** Lucy S. Witt, Radhika Prakash-Asrani, K. Ashley Jones, C. Christina Mehta, Zanthia Wiley, Jesse T. Jacob, Hasan F. Shabbir, Julianne Gent, Chad Robichaux, Jessica Howard-Anderson, Sujit Suchindran, Julia E. Szymczak, Raymund B. Dantes, Scott K. Fridkin

**Affiliations:** 1 Department of Medicine, Division of Infectious Diseases, Emory University School of Medicine, Atlanta, Georgia; 2 Department of Pharmacy, Emory Healthcare, Atlanta, Georgia; 3 Department of Medicine, Division of Hospital Medicine, Emory University School of Medicine, Atlanta, Georgia; 4 Office of Quality, Emory Healthcare, Atlanta, Georgia; 5 Department of Biomedical Informatics, Emory University, Atlanta, Georgia; 6 Department of Internal Medicine, Division of Epidemiology, University of Utah School of Medicine, Salt Lake City

## Abstract

**Importance:**

Antibiotic overuse is harmful to patients and the health care system. Antibiotic prescribing report feedback has been described in outpatient and long-term care settings but is relatively untested in inpatient settings.

**Objective:**

To assess the association between peer comparative inpatient antibiotic prescribing feedback and changes to antibiotic prescribing rates among hospitalists in a large health care network.

**Design, Setting, and Participants:**

This quality improvement study linked data on billing and prescribing of broad-spectrum antibiotics for hospital-onset infections (BS-HO antibiotics) from hospitalists at 5 diverse acute care facilities encompassing both academic and primarily community-based hospitals within the same large health care network. Data were pooled into 12 two-month periods from January 1, 2023, through December 31, 2024, in a quality improvement intervention with a stepped-wedge cluster design. Hospitalists who contributed billed patient days for at least one 2-month period during the study period received the intervention.

**Interventions:**

Observed-to-expected ratios (OERs) for days of therapy (DOT) of prescribed antibiotics were calculated for each hospitalist per period and reported back to hospitalists in a peer comparative report disseminated via email every 2 months.

**Main Outcomes and Measures:**

Hospitalists’ prescribing rates for BS-HO antibiotics with activity against *Pseudomonas aeruginosa* were assessed, accounting for time, clustering of hospitalists, and patient characteristics including comorbidities and clinical syndrome.

**Results:**

The study included 169 hospitalists (median per hospital, 30 [range, 24-50]) for a total of 1687 bimonthly observation periods. Per 2-month period, hospitalists had a mean (SD) of 126 (48) patient encounters. Among hospitalists at facilities receiving the intervention, the prescribing rate ratio (RR) for DOT was higher among clinicians caring for higher proportions of patients with sepsis (RR, 1.04; 95% CI, 1.00-1.08) and end-stage kidney disease (RR, 1.09; 95% CI, 1.05-1.14) and was lower for each sequential reporting period (RR, 0.99; 95% CI, 0.98-1.00). In multivariable models accounting for these variables and the trend over sequential periods, the intervention was not significantly associated with lower prescribing rates (RR, 0.97; 95% CI, 0.91-1.04).

**Conclusions and Relevance:**

Peer comparative inpatient prescribing reports for hospitalists were not associated with a change in hospitalists’ prescribing rates of BS-HO antibiotics. The findings suggest additional efforts to augment the utility of the reports are justified.

## Key Points

**Question:**

Can peer comparison feedback about broad-spectrum antibiotic orders decrease hospitalists’ prescribing of these antibiotics?

**Findings:**

In this quality improvement study among 169 hospitalists, clinician-specific feedback using peer benchmarking was not associated with subsequent changes to hospitalist prescribing of broad-spectrum antibiotics with activity against *Pseudomonas aeruginosa* when preexisting changes in prescribing over time were included in multivariable analysis.

**Meaning:**

In this study, peer comparison benchmarking for hospitalist prescribing of broad-spectrum antibiotics was not associated with prescribing habits beyond a concurrent, preexisting decrease in prescribing; however, the findings suggest this intervention could be optimized for improved efficacy.

## Teaser

This quality improvement study assesses whether an intervention providing peer comparison feedback about orders for broad-spectrum antibiotics was associated with decreased antibiotic prescribing among hospitalists in a large health care network.

## Introduction

Inappropriate antibiotic prescribing in both primary care and inpatient settings contributes to antibiotic resistance and associated adverse outcomes such as *Clostridioides difficile* (*C difficile*) infection and increased health care costs.^1,2^ While antibiotic stewardship interventions such as clinician education and feedback have shown promise in ambulatory settings, their translation to inpatient care faces unique challenges. Established inpatient prescribing metrics, such as the Standardized Antimicrobial Administration Ratio used by the Centers for Disease Control and Prevention’s (CDC) National Healthcare Safety Network (NHSN), lack the granularity to attribute prescribing patterns to individual clinicians or even specialties, focusing only on patient location.^3,4,5^ Furthermore, inpatient prescribing is often empiric, complex, and influenced by patient comorbidities and health care exposures, making benchmarking difficult.^3,6,7^ Addressing these limitations requires sophisticated, clinician-informed metrics. Our group previously developed a patient risk–adjusted metric for comparing hospitalist antibiotic prescribing.^8^ Building on this, our hospital medicine leadership and antimicrobial stewardship research team collaborated on a quality improvement initiative to reduce hospitalists’ excessive prescribing of empiric antibiotics for *Pseudomonas aeruginosa* activity. The current article summarizes the implementation strategy for providing peer comparative feedback reports to hospital medicine faculty in our health care network and the association of these reports with hospitalists’ prescribing practices.

## Methods

This quality improvement study conformed to the Standards for Quality Improvement Reporting Excellence (SQUIRE) 2.0 guidelines for reporting new work to improve health care safety.^9^ The Emory University institutional review board approved this study with expedited review, with the Health Insurance Portability and Accountability Act authorization and informed consent requirement waived because the study was determined to pose minimal risk.

### Context

Emory Healthcare is a multihospital network serving the Atlanta, Georgia, metropolitan area; hospital medicine faculty provide care for patients at 2 larger academic hospitals and 3 smaller community hospitals and care for a variety of patient types (eTable 1 in Supplement 1). Hospital medicine staffing models are similar at each hospital, with multiple nocturnists at each facility; advanced practice providers (APPs) provide daily follow-up care only at the 2 larger facilities. Although antibiotics may be initiated by the emergency department clinicians and propagated by admitting hospitalists, we attributed antibiotics administered to any patient on a date billed by a specific hospitalist (regardless of APP care or infectious disease consultation) to the billing hospitalist. Nocturnists were not targeted for intervention and were not included in the analysis.

All the included hospitals had antibiotic stewardship teams that harmonized efforts across facilities. These teams design and implement their programs using the Clinical Performance Feedback Intervention Theory (CP-FIT).^10^ This framework guides goal setting, prioritizing stewardship opportunities by achievability, patient safety impact, and evidence base. The intervention was timely due to similarly timed systemwide guidelines recommending specific treatments for urinary tract infections (UTIs) and pneumonia, was well received by stakeholders, aligned with data collection, was deemed clinically credible, and was distinct from concurrent stewardship efforts focused on methicillin-resistant *Staphylococcus aureus* nares screening to decrease unnecessary vancomycin use in patients with pneumonia.

### Intervention

The intervention comprised 2 parts: (1) a 1-time educational session on the report and antibiotic deescalation and (2) bimonthly email prescribing reports. Educational sessions, led by stewardship leads, focused on evidence-based recommendations for presumed community-acquired pneumonia and UTI, specifically indications for empiric antipseudomonal coverage, and use of Emory Healthcare’s antibiotic prescribing assistance tool. Feedback reports reinforced these indications and provided a link to the tool (eFigure 1 in Supplement 1). Hospitalist-specific feedback reports used observed-to-expected ratios (OERs), derived in part from EPIC Clarity System clinical and billing data (EPIC Systems Corporation). Generalized linear models generated OERs adjusted for proportions of patients with sepsis, end-stage kidney disease (ESKD), and UTIs. Although antibiotic choice is influenced by many patient factors, our group’s previous work identified these 3 risk factors as consistently associated with prescribing of broad-spectrum antibiotics for hospital-onset infections (hereafter, *BS-HO antibiotics*) (eTable 2 in Supplement 1).^8,11,12^ Every 2 months, the research team automatically emailed individualized PDF reports to each hospitalist. This intervention was not integrated into the electronic health record (EHR) to avoid pop-up fatigue. Report content and formatting considered CP-FIT’s key feedback elements, assessed through cognitive interviews with 13 hospitalists during the development phase, with corrective changes based on structured questions (eTable 3 in Supplement 1).

### Study Design

**Figure 1.**
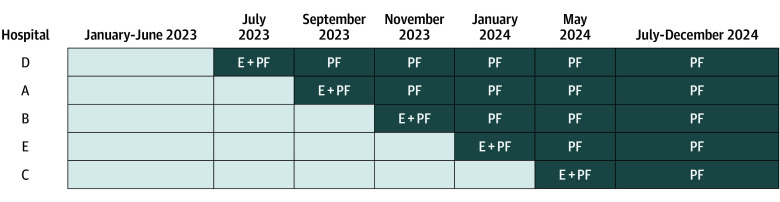
Chart of Stepped-Wedge Interventional Study Design Shading represents intervention periods with bimonthly hospitalist feedback (PF) reports. E indicates educational session.

A quality improvement intervention using a stepped-wedge cluster study design assessed the impact of prescribing reports. Of the 5 hospitals included with hospital medicine services (HMS), 4 shared an EHR, enabling randomization for the outcome assessment. The fifth hospital (hospital C) adopted the EHR at a later stage and joined after study initiation. The intervention was implemented bimonthly, 1 cluster at a time (Figure 1). Following baseline (January 1 to June 30, 2023), the first cluster began the intervention on July 1, 2023. The final cluster transitioned by March 1, 2024, with the intervention continuing through December 31, 2024. The study protocol was created in March 2022 and registered with ClinicalTrials.gov (NCT07368660) in January 2026 (Supplement 2). This study was registered late, as we interpreted our study as quality improvement research; however, after discussion with journal editors, we agreed that registration was appropriate.

### Implementation Assessment

One week after the third prescribing report was sent (6 months after the intervention began), the antibiotic stewardship team emailed a single-question survey to confirm whether recipients had viewed the reports. Hospitalists who did not respond received email reminders from the HMS faculty liaison, who then followed up with any remaining nonresponders.

### Study Measures

Data from January 2023 through December 2024 were extracted for inpatients receiving care from HMS at all hospitals. For each hospitalist, billing records were used to identify patient encounters and calculate billed patient-days (bPDs). Antibiotic prescribing data from electronic medication administration records captured days of antibiotic therapy (DOT) for NHSN-defined BS-HO agents per bPD. Patient characteristics linked to each encounter included age, microbiology results (bacteremia), *International Statistical Classification of Diseases and Related Health Problems, Tenth Revision (ICD-10)*–based antibiotic indications (eg, pneumonia, COVID-19, sepsis, and UTI), and comorbidities, enabling Elixhauser comorbidity calculation. Generalized linear models for hospitalist OERs are summarized in eTable 4 in Supplement 1. In each model, bPDs remained continuous, while other variables were categorized (defining a categorical high vs low exposure based on the quintile of exposure magnitude at which the association became significant). HMS leadership and focus groups preferred hospital-specific models to calculate OERs to maximize credibility.

#### Primary Outcome

The primary outcome was the observed hospitalist-specific antibiotic prescribing rate, defined as billed DOT of BS-HO antibiotics per 1000 bPDs. This measure alone was not risk adjusted; however, we adjusted for patient and hospitalist characteristics and included these as covariates in the regression model. For descriptive purposes, risk-adjusted hospitalist-specific OERs were estimated as mean values and plotted over time by facility.

#### Secondary Outcomes and Safety Metrics

Safety metrics and secondary outcomes were considered at the patient encounter level, including *C difficile* infection occurring during the inpatient stay or within 8 weeks after discharge, 30-day readmission (for any reason), in-hospital death, and prolonged length of initial hospital stay (defined as >7 days). To maximize the detection of safety indicators, the secondary outcome analysis was performed among all patients receiving care from the HMS and discharged with an *ICD-10* code indicating community-acquired pneumonia or UTI. During interim analysis, all in-hospital deaths were reviewed (L.S.W., S.S.) to ascertain any association with deescalation of antibiotics.

### Statistical Analysis

There was natural churn of hospitalists leaving and entering the health care network; we included hospitalists with at least one 2-month hospitalist period, regardless of when this occurred relative to the intervention. Comparisons between density of hospitalist patient care, types of patients, and rates of antibiotic use were compared between the preintervention and intervention periods. For the primary outcome analysis, we used a generalized mixed-effects model with a negative binomial distribution to account for overdispersion and a log link function to evaluate the association between the intervention and antibiotic prescribing rates over time. The observed DOT measure was modeled on the hospitalist-period level with an offset for patient-days to control for secular trends and a random effect for facility to account for clustering of hospitalists within facilities. A random intercept for each hospitalist nested within facility was included to account for correlation among repeated observations within hospitalists and the hierarchical structure of the data. The intracluster correlation coefficient (ICC) was estimated to quantify the proportion of total variance attributable to differences between clusters. In addition, the model adjusted for patient mix characteristics as covariates.^13^ The main analysis was limited to the 4 hospitals included in the initial study protocol (approved in March 2022). Sensitivity analyses of the primary outcome were performed using only hospitalists having indicated they were receiving and reading the reports to account for implementation bias. An additional sensitivity analysis was completed evaluating the primary outcome including the fifth hospital, which was added after study initiation.

Secondary outcomes were analyzed at the patient level for all patients having received any care by the HMS and with a discharge code for UTI or community-acquired pneumonia. Safety analysis used generalized mixed-effects regression models using a logit link function to evaluate the association of the intervention with each outcome over time. The model accounted for repeat hospitalizations within patients and for clustering within facility. Covariates also included time (modeled categorically given the nonlinear trend in outcomes) and patient indicators (COVID-19, influenza, Elixhauser comorbidity, and sepsis). All analyses were performed using R, version 4.2.0 (R Project for Statistical Computing), and 1-sided *P* values less than .05 were considered significant.

## Results

### Study Sample Description

Across the 5 hospitals, 169 hospitalists (median per facility, 30 [range, 24-50]) contributed to 1687 bimonthly observations (716 [42.4%] preintervention and 971 [57.6%] during the intervention) during the 24-month study period and a total of 541 401 bPDs across all hospitals (42.8% preintervention and 57.2% during the intervention) (Table 1). Per 2-month period, hospitalists had a mean (SD) of 126 (48) patient encounters. There were small variations in patient mix and prescribing metrics between the preintervention and intervention periods, with slight reductions in the median prescribing rate but similar median bPDs. The number of hospitalist periods (observations) contributed per hospitalist ranged from 1 to 12, with 96 hospitalists (56.8%) included in all periods and another 49 (29.0%) included in 6 to 11 periods, 17 (10.1%) in 2 to 5 periods, and only 4 (2.4%) in a single period. Because 1 hospital (hospital C) was not yet part of our consolidated network at the time of study initiation, data from those 24 hospitalists (14.2%) were not included in the primary outcome analysis, which included the remaining 145 hospitalists (85.8%).

**Table 1.** Hospitalist Care, Patient Demographics, and Illness Types, by Intervention Period

Characteristic^a^	Study period	
	Preintervention	Intervention
Total bimonthly observations, No. (%) (N = 1687)	716 (42.4)	971 (57.6)
Billed patient-days		
Total, No. (%) (N = 541 401)	231 945 (42.8)	309 456 (57.2)
Per hospitalist period, median (IQR)	328 (239-400)	330 (231-392)
By patient attribute, median (IQR), %		
Age ≥65 y	52 (46-58)	52 (44-61)
Pneumonia	6 (4-8)	5 (3-7)
COVID-19	3 (1-6)	2 (1-5)
Sepsis	7 (5-10)	6 (4-9)
Urinary tract infection	11 (8-14)	9 (7-12)
End-stage kidney disease	9 (5-13)	7 (5-11)
Malignant neoplasm	13 (9-17)	12 (9-15)
Neurologic disorder	11 (8-14)	10 (7-13)
Obesity	18 (14-23)	18 (15-22)
Charlson Comorbidity Index score >2^b^	83 (78-87)	78 (73-83)
Broad-spectrum agents for hospital-onset infections per period		
Days of therapy, median (IQR)		
Overall	34 (23-48)	30 (19-45)
Per 1000 patient-days	113 (81-149)	104 (73-135)
Ratio of observed to expected	0.98 (0.76-1.20)	0.88 (0.68-1.11)
Patients with pneumonia or urinary tract infection, No. (%) (n = 15 350)	6273 (40.9)	9077 (59.1)
Readmission within 30 d	1172 (18.7)	1718 (18.9)
Length of stay >7 d	2341 (37.3)	3439 (37.9)
*C difficile* infection	99 (1.6)	189 (2.1)
In-hospital death	101 (1.6)	157 (1.7)

Abbreviation: *C difficile*, *Clostridioides difficile*.

^a^ Patient characteristics, including diagnosis of pneumonia or urinary tract infection, were ascertained through established *International Statistical Classification of Diseases and Related Health Problems, Tenth Revision, *codes from discharge.

^b^ Total score range, 0 to 37, with scores greater than 2 considered as having a high burden of comorbidities and increased risk of 10-year mortality.

### Prescribing Rates, Process Measures, and Context

Systemwide, the rate of prescribing BS-HO antibiotics varied greatly between hospitals at the start of the study period and trended downward at all facilities, with a reduction in variability between facilities at end of study (eFigure 2 in Supplement 1). The most used BS-HO antibiotics prescribed were piperacillin-tazobactam, meropenem, and ceftazidime. Only 94 of the 145 hospitalists included in the primary outcome analysis (64.8%) responded to the 1-question survey; of those, 6 respondents (6.4%) did not recall receiving reports, and during one-on-one calls with investigators they were directed to subject line and sender information, ultimately locating the reports to facilitate viewing. Another 9 (9.6%) did not understand the reports and were educated during one-on-one calls with their local stewardship lead. The remaining 76 (80.9%) confirmed receipt and understanding of reports and were included in the sensitivity analysis.

### Primary Outcomes

#### OER Trends, Regression, and Sensitivity Analysis

**Figure 2.**
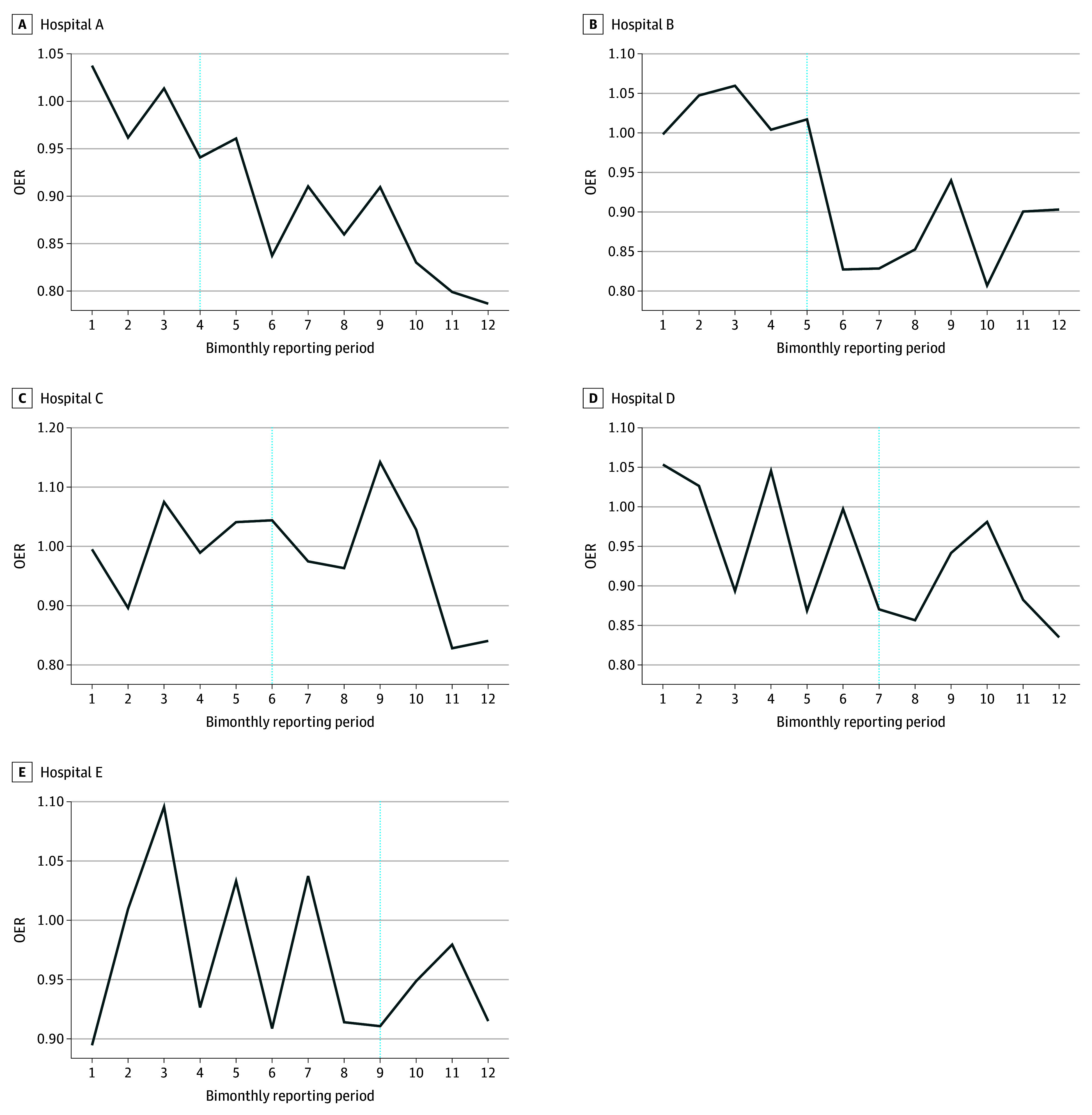
Line Graphs Showing Observed-to-Expected Ratio (OER) of Antibiotic Use Over the 24-Month Study Period at Each Hospital, Reported as 12 Bimonthly Observations Vertical lines represent the start of the intervention.

Over the study period, the OER was normally distributed at each facility, with slight differences between facilities (median OER, 0.93 [range, 0.90-0.94]). The mean OER trended downward over time, with considerable variability between reporting periods and a continued downward trend after the intervention began (Figure 2). In the primary analysis limited to the 4 initially enrolled facilities, 145 hospitalists contributed 1444 bimonthly periods included in the regression analysis. The crude model estimated the prescribing rate ratio (RR) during the intervention phase at 0.92 (95% CI, 0.87-0.96); however, in the fully adjusted model accounting for time and patient factors, the intervention was not significant (prescribing RR, 0.97; 95% CI, 0.91-1.04), indicating no significant association of the intervention with the primary outcome (Table 2). Hospitalists caring for more patients with sepsis (>6.2% of patient-days with sepsis) prescribed at higher rates (prescribing RR, 1.04; 95% CI, 1.00-1.08), as did hospitalists caring for more patients with ESKD (>10.4% of patient-days), with a prescribing RR of 1.09 (95% CI, 1.05-1.14). Each subsequent reporting period was also associated with a slightly lower prescribing rate (prescribing RR, 0.99; 95% CI, 0.98-1.00). Caring for more patients with UTI (>12.2% of patient-days) was not significantly associated with a difference in prescribing rate (prescribing RR, 1.00; 95% CI, 0.97-1.05) (Table 2 and eTable 4 in Supplement 1).

**Table 2.** Regression Estimates for the Association Between the Intervention and Days of Therapy of BS-HO Attributed to Hospitalists in Both Crude and Adjusted Models

Variable^b^	Prescribing RR (95% CI)^a^	
	Crude model	Fully adjusted model
Intercept	0.11 (0.11-0.12)^c^	0.11 (0.09-0.15)^c^
Intervention	0.92 (0.87-0.96)^c^	0.97 (0.91-1.04)
Time, per subsequent reporting period	NA	0.99 (0.98-1.00)^d^
Sepsis	NA	1.04 (1.00-1.08)
Urinary tract infection	NA	1.00 (0.97-1.05)
End-stage kidney disease	NA	1.09 (1.05-1.14)^c^

Abbreviations: BS-HO, broad-spectrum antibiotics for hospital-onset infections; NA, not applicable; RR, rate ratio.

^a^ The 95% CIs were calculated using the glmmTMB function in R, version 4.2.0 (R Project for Statistical Computing). Fixed-effect coefficients were estimated on the log-link scale, and RRs were obtained by exponentiation, with 95% CIs calculated using the Wald (normal-approximation) method based on the model-based SEs of the fixed effects.

^b^ All variables were identified by *International Statistical Classification of Diseases and Related Health Problems, Tenth Revision, *codes.

^c^ *P* < .001.

^d^ *P* < .05.

The total variance (σ^2^) among 145 hospitalists in 4 hospitals across 1444 observations was 2.28. The ICC was 0.04, indicating that 4% of the variation in the outcome was attributable to clustering at the hospitalist and facility level. Although the ICC was only 0.04, the conditional *R*^2^ was higher than the marginal *R*^2^ (0.045 vs 0.002), indicating that nearly all explained variance was attributable to between-hospitalist (τ_00_ = 0.03) or between-facility (τ_00_ = 0.07) differences rather than individual fixed effects. This suggests that group-level variation played a small role and highlights the limited explanatory power of the measured covariates; most variation in the prescribing rate remained unexplained.

A sensitivity analysis including only the 76 hospitalists who confirmed both receipt and understanding of prescribing reports (52.4% of the 145 included in the primary outcome analysis) yielded similar results for the association and statistical significance. A second sensitivity analysis used an expanded group of hospitalists at all 5 facilities; no appreciable changes were observed (eTable 5 in Supplement 1).

### Secondary Outcomes and Safety Analysis

The secondary outcome analysis was limited to 15 350 patients having received care by any hospitalist and with an *ICD-10*–coded discharge consistent with UTI or pneumonia; of these, 2890 (18.8%) were readmitted for any reason within 30 days, 258 (1.7%) died while in the hospital, 288 (1.9%) developed *C difficile* infection during hospitalization or within 8 weeks of hospitalization, and 5780 (37.7%) had a length of hospital stay longer than 7 days (Table 1). No significant difference in each of the outcomes evaluated was observed (Table 3). In contrast, 2 patient factors, sepsis and higher Elixhauser score, were associated with poorer outcomes (Table 3).

**Table 3.** Association of the Underlying Illness and the Intervention With Secondary Outcomes Among Inpatient Encounters With Either Pneumonia or Urinary Tract Infection Diagnosis During the Study Period

Factor	AOR (95% CI)^a^			
	Length of stay >7 d	30-d Readmission	In-hospital death	*C difficile* infection
Intervention	1.06 (0.90-1.26)	0.90 (0.75-1.08)	1.69 (1.00-2.85)	0.81 (0.40-1.65)
COVID-19	1.04 (0.91-1.20)	0.89 (0.74-1.06)	1.40 (0.92-2.11)	0.72 (0.32-1.63)
Influenza	0.77 (0.58-1.03)	0.71 (0.49-1.04)	0.88 (0.32-2.37)	0.78 (0.17-3.60)
Sepsis	4.61 (3.92-5.42)	1.04 (0.87-1.23)	4.17 (3.14-5.55)	2.85 (1.68-4.82)
Elixhauser score	1.02 (1.02-1.02)	1.01 (1.01-1.01)	1.02 (1.01-1.02)	1.00 (1.00-1.01)

Abbreviations: AOR, adjusted odds ratio; *C difficile*, *Clostridioides difficile*.

^a^ The AOR for each outcome—attributed to a patient with an *International Statistical Classification of Diseases and Related Health Problems, Tenth Revision, *code for each patient factor, adjusting for each other factor, for each unit increase in the Elixhauser comorbidity score—was associated with a 2% increase in the odds of prolonged length of stay (>7 days), 1% increase in odds of 30-day readmission, 2% increase in death, and no increase in odds of *C difficile* infection.

## Discussion

This report summarizes the systematic implementation of a stepped-wedge cluster design quality improvement intervention using a peer comparative antibiotic prescribing metric to change the antibiotic prescribing behavior of hospitalists across 5 acute care hospitals during a 24-month period. Distribution of the personalized report was not associated with a reduction in prescribing rates of the CDC NHSN’s BS-HO antibiotic group beyond preexisting downward trends. Although these results may be viewed as a negative study outcome, there are several important findings that can help direct stewardship efforts aimed at hospitalists using peer comparative prescribing data.

First, this study used a stepped-wedge design. This design avoids the pitfalls of similar quality improvement initiatives using other designs, such as before-and-after studies, which are known to be confounded by temporal trends, or controlled before-and-after studies, subject to other confounding biases.^13^ Second, the rollout was assigned agnostically to minimize bias by local champions of the effort who may have advocated for change early in the study. The findings were consistent across 5 very different hospitals, although confined to a single health care network. In relation to both these points, the study design and statistical approach accounted for temporal trends and nonindependence of observations; if these data were analyzed as a before-and-after intervention study, assuming independence of observations, we would have detected significant reductions associated with the intervention (Table 1). A third attribute is the care taken to assess the penetration of the intervention through an implementation check; because a subanalysis limited to only hospitalists acknowledging reading of the report showed no appreciable differences in the results, we do not consider poor penetration of the reports as a reason for the lack of association, as reported as a limitation in studies of peer comparative communication.^10,14,15^ Fourth, the design of the report was the result of an iterative process with the study’s HMS leadership team through cognitive interviews to consider pathways of feedback outlined in CP-FIT theory.^10^ The cognitive interviews were valuable in refining the design and content of the reports for readability, comprehension, and method of delivery. These strengths all minimize the threats to internal validity usually associated with studies using experiments in which the intervention is not randomly assigned.^16^

Furthermore, efforts were made to ensure credibility of the OER as a prescribing metric, including dedicated analysis to quantify the association of infectious disease consultation with individual hospitalists’ OERs and the persistence of clinicians with high antibiotic prescribing rates to continue to prescribe at high rates during subsequent periods.^17,18^ Stewardship teams did not proactively engage clinicians with high antibiotic prescribing rates to review prescribing, but this has been proposed as a potential modification for ongoing use. If proactive engagement had been part of the study design, we anticipate the impact of the intervention would have been significant. Notably, the statistical results of the intervention were unexpected; we had not anticipated that rates of prescribing would decrease across most facilities during the preintervention period, likely muting the impact of the intervention.

We believe the framework for influencing inpatient prescriber behavior through peer comparative feedback as outlined in the relevant literature is solid and should result in practice change.^10,12,15^ There are few peer comparative feedback studies in acute care hospitals, and efforts in long-term care previously studied^15^ were more impactful than in our study. This may relate to the complexity of care among acute care patients on hospitalists’ service, where deescalation is both a concern and a focus, compared with avoiding unnecessary antibiotic initiation among nursing home residents.^14,15^ Also, our choice of emailing a PDF document requiring steps to visualize the data likely influenced our results. Only 6.4% of the hospitalists responding to our implementation check did not notice the emails or attachments, similar to what has been reported in the outpatient setting.^15,19,20^

Launching this initiative was costly, including data scientist and engineering time to curate data tables within the clinical and billing data; validate extraction and linkage to billing data; and design, develop, and create bimonthly reports. Considering the equivocal influence of the peer comparative reports alone, we would not recommend continuing this effort without modification. Alternative implementation strategies could include targeted outreach by stewardship teams to clinicians with repeatedly high prescription rates or the development of an EHR-based template.

### Limitations

Our study has multiple limitations. Our health care system has a unified division of hospital medicine, which may have led to discussion of our intervention among HMS hospitalists prior to its being formally introduced at their specific facility. We also did not assess how frequently hospitalists reviewed their reports or the timing of their review. In addition, our own modeling efforts suggested that significant variation in prescribing was not accounted for by our chosen variables, implying that unmeasured variables may affect prescribing.

## Conclusions

In this quality improvement study, we found that peer benchmarked prescribing reports of BS-HO antibiotics were not associated with a reduction in hospitalist prescribing of these antibiotics. The intervention should be sustainable for programs with similar adherence to EHR standards; however, the findings suggest more engagement with hospitalists by stewardship teams may be required to ensure credibility and encourage best practices.

## Notes

Supplement 1. **eTable 1.** Approximate Participating Hospital Characteristics and Patient Population Distribution in 2022 **eTable 2.** Broad-Spectrum Hospital Onset Antibiotics as Defined by National Healthcare Safety Network **eTable 3.** Cognitive Interviews With 13 Hospital Medicine Providers Regarding Clinical Performance Feedback Intervention and Draft Report Design Considerations and Design Fault Mitigation Strategies **eTable 4.** Parameter Estimates for Linear Regression Models Predicting Days of Therapy of Broad-Spectrum Hospital-Onset Antibiotics, by Facility and Overall **eTable 5.** Negative Binomial Mixed Effects Regression Estimating the Association of the Intervention With DOT of BS-HO Antibiotics Attributed to Hospitalists **eFigure 1.** Example of Final Provider-Specific Prescribing Report **eFigure 2.** Rate of Broad-Spectrum Hospital-Onset Antibiotic Use (Days of Therapy per 1000 Patient-Days) at Each Hospital

Supplement 2.
Study Protocol

Supplement 3.
Data Sharing Statement
