# Supplementary material for: Association Between Peer Comparison Feedback and Hospitalist Antibiotic Prescribing
Source: JAMA Netw Open. 2026 Apr 28;9(4):e269504. doi: 10.1001/jamanetworkopen.2026.9504 (PMC13126217; doi:10.1001/jamanetworkopen.2026.9504)
Supplement: Supplement 1. — eTable 1. Approximate Participating Hospital Characteristics and Patient Population Distribution in 2022 eTable 2. Broad-Spectrum Hospital Onset Antibiotics as Defined by National Healthcare Safety Network eTable 3. Cognitive Interviews With 13 Hospital Medicine Providers Regarding Clinical Performance Feedback Intervention and Draft Report Design Considerations and Design Fault Mitigation Strategies eTable 4. Parameter Estimates for Linear Regression Models Predicting Days of Therapy of Broad-Spectrum Hospital-Onset Antibiotics, by Facility and Overall eTable 5. Negative Binomial Mixed Effects Regression Estimating the Association of the Intervention With DOT of BS-HO Antibiotics Attributed to Hospitalists eFigure 1. Example of Final Provider-Specific Prescribing Report eFigure 2. Rate of Broad-Spectrum Hospital-Onset Antibiotic Use (Days of Therapy per 1000 Patient-Days) at Each Hospital [file jamanetwopen-e269504-s001.pdf]

Supplementary Online Content

Witt LS, Prakash-Asrani R, Jones KA, et al. Association between peer comparison feedback and hospitalist antibiotic prescribing. *JAMA Netw Open*. 2026;9(4):e269504. doi:10.1001/jamanetworkopen.2026.9504

eTable 1. Approximate Participating Hospital Characteristics and Patient Population Distribution in 2022

eTable 2. Broad-Spectrum Hospital Onset Antibiotics as Defined by National Healthcare Safety Network

eTable 3. Cognitive Interviews With 13 Hospital Medicine Providers Regarding Clinical Performance Feedback Intervention and Draft Report Design Considerations and Design Fault Mitigation Strategies

eTable 4. Parameter Estimates for Linear Regression Models Predicting Days of Therapy of Broad-Spectrum Hospital-Onset Antibiotics, by Facility and Overall

eTable 5. Negative Binomial Mixed Effects Regression Estimating the Association of the Intervention With DOT of BS-HO Antibiotics Attributed to Hospitalists

eFigure 1. Example of Final Provider-Specific Prescribing Report

eFigure 2. Rate of Broad-Spectrum Hospital-Onset Antibiotic Use (Days of Therapy per 1000 Patient-Days) at Each Hospital

This supplementary material has been provided by the authors to give readers additional information about their work.

© 2026 Witt LS et al. *JAMA Network Open*.

eTable 1: Approximate participating hospital characteristics and patient population distribution in 2022

Hospital	Characteristics	Bed Count	Primarily Academic or Community	% African American	% Hispanic	% Without a Primary care physician	% Uninsured
Hospital A	Suburban, non-profit	582	Academic	46.1%	5.2%	40.1%	4.7%
Hospital B	Urban, non-profit	537	Academic	71.6%	3.1%	53.6%	6.4%
Hospital C	Suburban, non-profit	451	Community	50.4%	5.5%	48.3%	5.9%
Hospital D	Suburban, non-profit	373	Community	32.4%	5.8%	43.7%	3.9%
Hospital E	Suburban, non-profit	152	Community	19.7%	6.7%	36.8%	4.4%

© 2026 Witt LS et al. *JAMA Network Open*.

eTable 2. Broad-Spectrum Hospital Onset Antibiotics as Defined by National Healthcare Safety Network

Amikacin (Intravenous)	Gentamicin (Intravenous)
Aztreonam (Intravenous)	Imipenem/Cilastatin
Cefepime	Meropenem
Ceftazidime	Piperacillin/Tazobactam
Doripenem	Tobramycin (Intravenous)

© 2026 Witt LS et al. *JAMA Network Open*.

eTable 3. Cognitive interviews with 13 hospital medicine providers regarding clinical performance feedback intervention and draft report design considerations and design fault mitigation strategies

Design Step	Responses to structured queries regarding mock-up reports on prescribing metrics			Corrective Action to Report
	No. (%)	Summary Assessment	Relevant Subjective Comments or Notes	
Perceived practice habits and benefits of initiative	6 (45%)	Believe ≥1:3 patients are on antibiotics	"Lots of patients have multiple issues that can be treated by antibiotics“	Ensure report points to on-line guidance application; incorporate “antibiotic inertia” into educational session
	7 (53%)	Believe they often change antibiotics on plans initiated by ED	”I often change as more information becomes available, but can probably use help”	
	5 (38%)	Believe they rarely change antibiotics on plans initiated by ED	"We need access to a document [of anti-pseudomonal indications]…accessible daily"	
Seeing and reading of report	13 (100%)	Recognized report was on prescribing	Reading report took about 5-10 seconds; font and size issues were identified	Improved font size, reduced content
Comprehension of report	11 (84%)	Reflects personal prescribing	None	Removed 66% of the data values (those not commented on). Graphic for ranking improved, highlighted and simplified interpretation; excess prescribing value retained but moved to top with interpretation
	9 (69%)	Identified magnitude of antibiotics prescribed above “normal”	“I am not a data person…I am not sure where you want me to look”	
	7 (53%)	Accurate interpretation of the prescribing metric		
	4 (30%)	Identified relative ranking among peers correctly	“Ranking is easier to interpret, but the 35% gives a more quantitative value…I want both”	
	1 (10%)	Able to Interpret >1/2 of the data		
Acceptance (Credibility)	6 (45%)	Believe metric reflects ED prescribing more than hospitalists’ prescribing	“…need [in the report or other time] to instill confidence that the attribution of prescribing is linked to the right provider”	Commit to study on impact of infectious disease consultation on metric. Added visual clues to graphic highlighting target achievement
	7 (53%)	Believe metric is accurate	“…I would like to know what specific conditions I am over prescribing for…”	
	3 (23%)	Identified the target metric		
Forming intention to act	11 (84%)	Identified potential actions and focus on de-escalation	“…it was obvious which antibiotics its focused on, I get it, this is helpful”	Slight edits to infographic
Report delivery	11 (84%)	Prefer email to pop-ups in EHR	None	None

Notes: ED, Emergency Department; EHR, electronic health record.

© 2026 Witt LS et al. *JAMA Network Open*.

eTable 4. Parameter estimates (Est) for linear regression models predicting days of therapy of broad-spectrum hospital-onset antibiotics, by facility and overall

	Facility											
	System-wide		A		B		C		D		E	
Parameter	Est	P-value	Est	P-value	Est	P-value	Est	P-value	Est	P-value	Est	P-value
(Intercept)	1.88	0.36	6.8	0.81	3.26	0.14	2.1	0.48	0.18	0.97	-2.6	0.50
No. billed patient-days (bPD)	0.10	<.001	0.1	<.001	0.06	<.001	0.2	<.001	0.14	<.001	0.1	<.001
> 6.2 % of bPD with Sepsis	2.44	0.04	9.5	0.08	0.08	0.95	-1.8	0.43	3.81	0.16	6.5	0.01
>12.2 % bPD with UTI	1.03	0.34	6.66	0.28	-0.86	0.62	-0.6	0.73	2.7	0.30	-0.2	0.92
>10.4 % bPD with ESRD	0.77	0.50	6.2	0.39	2.41	0.09	2.0	0.40	7.2	0.04	1.9	0.53
**Numbers included**	**System-wide**		**A**		**B**		**D**		**E**		**C**	
Providers	154		36		42		29		23		24	
Observations	573		126		158		112		89		88	

Note: Est, Parameter Estimate; percents listed are for high percentage of patient-days with the characteristics (top 60th to 100th percentile for UTI and ESRD and top 40th – 100th percentile for Sepsis).

Equation for calculating predicted: days of therapy = intercept + (bpd_coeff*bpd value for X provider) + (sepsis_coeff*sepsis_c value for X provider) + (uti_coeff*uti_c value for X provider) + (esrd_coeff*esrd_c value for X provider)

© 2026 Witt LS et al. *JAMA Network Open*.

eTable 5 Negative Binomial Mixed Effects Regression estimating the association of the intervention with DOT of BS-HO antibiotics attributed to Hospitalists

	Crude Model		Fully Adjusted Model		Fully Adjusted Model Including Fifth, Non-randomized Hospital		Fully Adjusted Model Including Providers Who Understood Report	
**Predictors**	**Incidence Rate Ratios**	**95% CI**	**Incidence Rate Ratios**	**95% CI**	**Incidence Rate Ratios**	**95% CI**	**Incidence Rate Ratios**	**95% CI**
(Intercept)	0.11 ***	0.11 – 0.12	0.11 ***	0.09 – 0.15	0.11 ***	0.09 – 0.14	0.11 ***	0.08 – 0.14
Intervention	0.92 ***	0.87 – 0.96	0.97	0.91 – 1.04	0.97	0.91 – 1.02	0.96	0.88 – 1.05
Time			0.99 *	0.98 – 1.00	0.99 *	0.98 – 1.00	0.99	0.98 – 1.00
Sepsis			1.04	1.00 – 1.08	1.03	1.00 – 1.07	1.07 **	1.02 – 1.13
UTI			1.00	0.97 – 1.05	1.01	0.97 – 1.05	1.03	0.98 – 1.09
ESRD			1.09 ***	1.05 – 1.14	1.08 ***	1.04 – 1.12	1.10 **	1.04 – 1.17
σ^2			2.28		2.28		2.3	
τ_{00}			0.03 id:entity		0.03 id:entity		0.03 id:entity	
			0.07 entity		0.06 entity		0.07 entity	
ICC			0.04		0.04		0.04	
N			145 id		169 id		72 id	
			4 entity		5 entity		4 entity	
Observations	1444		1444		1687		767	
Marginal R^2 / Conditional R^2	NA		0.002 / 0.045		0.002 / 0.039		0.003 / 0.042	

Notes: σ^2 Total Variance; τ_{00} Variance Components: Provider and Facility; ICC Intra-Cluster Correlation; N: Number of Providers and Number of Facilities. All variables identified by ICD-10 codes.

** p<0.05 ** p<0.01 *** p<0.001*

© 2026 Witt LS et al. *JAMA Network Open*.

eFigure 1. Example of final Provider-specific Prescribing Report

Your Personal 2-Month Antibiotic Prescribing Report – Since EPIC Transition Jan 2023

Month<br>Provider Name<br>Provider NPI<br>Primary Practice	

Your Performance
On par with peers :
0.75% less antibiotics than expected

*Risk adjusted, About **two-thirds** of your Hospital Medicine peers caring for patients with similar characteristics as yours prescribed fewer antibiotics. Prescribing less than expected should be an achievable target for you.

**Broad Spectrum Antibiotic Use**: Cefepime, Ceftazidime, Meropenem, Piperacillin/Tazobactam		
**Your Values**		
Patients	Percent your antibiotic orders exceed expected	
	Current Period	Previous 6 months
**158**	**-1%**	**-1%**

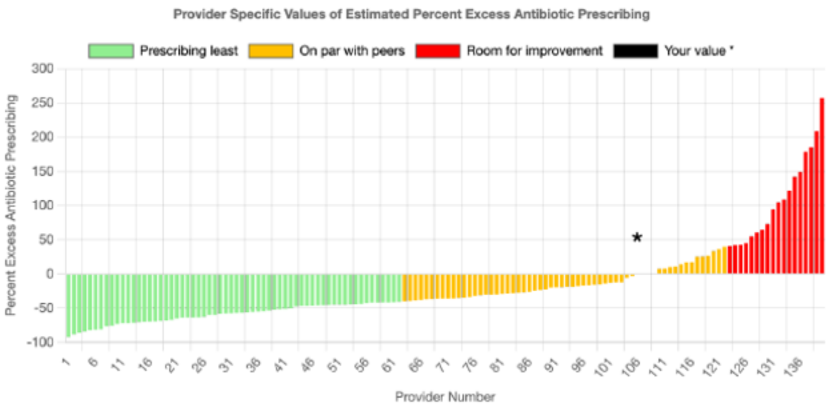

Key steps to improve your metrics and get to No Excess antibiotic use

Avoiding unnecessary broad-spectrum antibiotics is one of the most important actions you can take to help reduce antibiotic resistant and *C. difficile* infections. Actions for CAP, Urosepsis are:

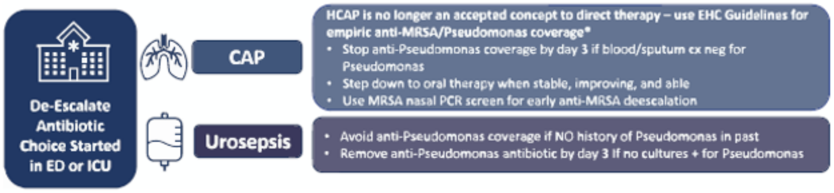

*Access EHC Antimicrobial Stewardship App when in VDT at: https://www.unboundmedicine.com/ucentral and click "register free/Emory" to download uCentral on mobile and use EHC login credentials

© 2026 Witt LS et al. *JAMA Network Open*.

eFigure 2. Rate of broad-spectrum hospital-onset antibiotic use (days of therapy per 1000 patient-days) at each hospital

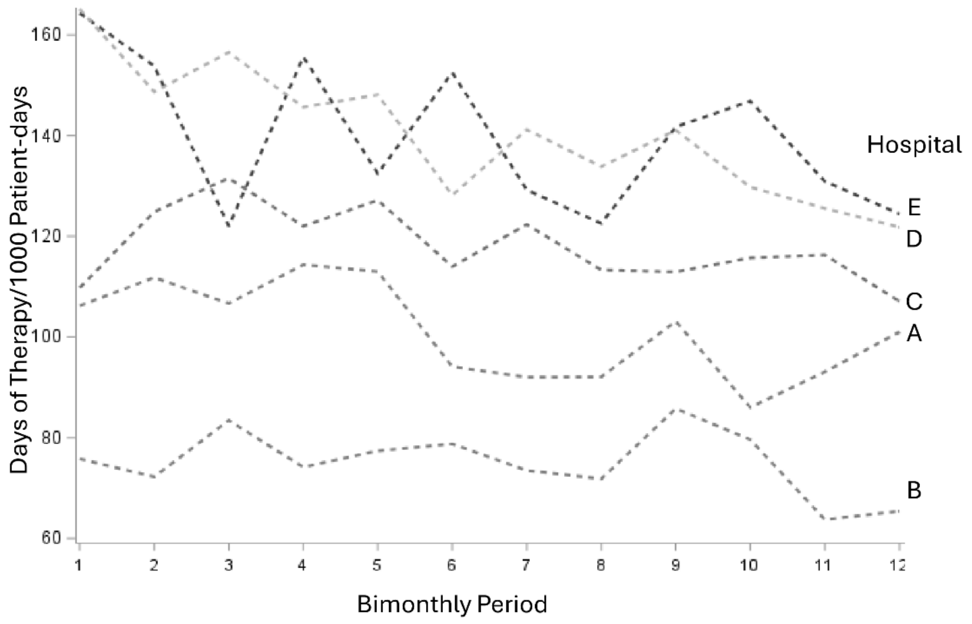

© 2026 Witt LS et al. *JAMA Network Open*.
