# Supplementary material for: Association Between Peer Comparison Feedback and Hospitalist Antibiotic Prescribing
Source: JAMA Netw Open. 2026 Apr 28;9(4):e269504. doi: 10.1001/jamanetworkopen.2026.9504 (PMC13126217; doi:10.1001/jamanetworkopen.2026.9504)
Supplement: Supplement 2. — Study Protocol [file jamanetwopen-e269504-s002.pdf]

PROTOCOL TITLE: InPART Rx - Inpatient Provider Antibiotic Rate Benchmarking to Reduce Unnecessary Prescribing

EXTERNAL (NON-EMORY) COLLABORATORS

None

PRINCIPAL INVESTIGATOR:

Scott K. Fridkin, MD

Department of Medicine, Division of Infectious Diseases

404-317-1164

sfridki@emory.edu

VERSION: 1

FUNDING SOURCE: Centers for Disease Control and Prevention (Grant # U54CK000601-01)

REVISION HISTORY

Revision #	Version Date	Summary of Changes
2	2/22/22	Incororated all clarifications, spelling.

Table of Contents

1. Study Summary 5
2. Objectives 6
3. Background 6
4. Studey Endpoints 7
5. Study Intervention/Investigational Agent 8
6. Procedures Involved 8
7. Analysis Plan and Outcomes. 9
8. Data Specimen Banking 10
9. Sharing of Results with Participants 10
10. Study Timelines 10
11. Inclusion and Exclusion Criteria 11
12. Population. 11
13. Vulnerable Populations 11
14. Local Number of Participants 11
15. Recruitment Methods 11
16. Withdrawal of Participants 12
17. Risk to Participants 12
18. Potential Benefits to Participants 12
19. Compensation to Participants 12
20. Data Management and Confidentiality 13
21. Provisions to Monitor the Data to Ensure the Safety of Participants 13
22. Provisions to Protect the Privacy Interest of Participants 13
23. Economic Burden to Participants 13
24. Informed Consent 13
25. Setting 14
26. Resources Available 14

27. Multi-Site Research When Emory is the Lead Site 14

28. References 15

Study Summary

**Project Title**	InPART Rx - Inpatient Provider Antibiotic Rate Benchmarking to Reduce Unnecessary Prescribing
**Project Design**	Phase 1: Retrospective analysis of summary encounter data by prescriber<br><br>Phase 2: Prospective evaluation of impact of quality-improvement activity composed of feeding back peer comparison data on antibiotic prescribing to prescribers
**Primary Objective**	Determine the impact of using an automated peer comparison of antibiotic prescribing on hospitalists' prescribing rates
**Secondary Objective(s)**	Determine variability and predictors of antibiotic prescribing by Hospital Medicine Service (HMS) across four Emory Healthcare Hospitals<br><br>Demonstrate no adverse effects on patients cared for by HMS during study period
**Research Intervention(s)/Interactions**	Provide messaging bi-monthly to hospitalists via email that contains peer comparison data and antibiotic prescribing educational content
**Study Population**	Primary population is all Hospital Medicine Service (HMS) Providers at EUH, EUHM, EJCH, ESJH.
**Sample Size**	Including 147 HMS providers, accounting for cluster effect we should be able to detect a large effect size (.9 to 1.1) with 80% statistical power. This translates to reductions in prescribing rates of 10% during intervention period from baseline period.
**Study Duration for individual participants**	October 2022 – December 2024

**Study Specific Abbreviations/ Definitions**	Hospital Medicine Service (HMS)<br>Standardized Antibiotic Prescribing Ratio (SAR)<br>C. difficile infection (CDI)<br>Days of Therapy (DOT)<br>Patient-days (PD)
**Funding Source (if any)**	Centers for Disease Control and Prevention

Objectives

Primary Objective: **Determine the impact of using an automated peer comparison of antibiotic prescribing on hospitalists' prescribing rates**

Secondary Objectives:

1) Demonstrate no adverse effects on patients cared for by HMS during study period
2) Determine variability and predictors of antibiotic prescribing by Hospital Medicine Service (HMS) across four Emory Healthcare Hospitals

Background

Approximately 50% of hospitalized patients receive an antibiotic during their stay.(A) Currently >75% of hospitals, including all highly ranked academic medical centers, employ hospitalists who increasingly provide care for inpatients and represent a prime target for inpatient antibiotic stewardship efforts.(B,C) Hospitalists care for newly admitted patients and those transitioning from critical and long term care, and frequently prescribe antibiotics for common inpatient problems (e.g., urinary tract infection, pneumonia). Recent studies of antibiotic prescribing data for these conditions suggest opportunities to improve prescribing accuracy: an estimated 30% of antibiotics used for these common infections were either unnecessary or prescribed incorrectly (D). In the ambulatory setting, there is substantial evidence that improving provider prescribing practices can be achieved by using peer comparisons of provider-specific antibiotic prescribing rates for benchmarking and feedback (E,F,G,H). Similarly, in the ambulatory setting Emory Healthcare (EHC) through a Quality Initiative (QI) has successfully used peer comparisons of clinic-specific and provider-specific prescribing metrics and targets (I).

The Study team has expanded this QI to the inpatient setting by exploring provider-specific prescribing metrics for EHC hospitalists. The goal of this QI work is to use traditional QI methods of peer-comparison through data feedback to improve antibiotic prescribing among EHC HMS by reducing unnecessary antibiotic use and improve patient safety on the

EHC HMS. This protocol outlines discrete steps taken to evaluate the impact of this expanded QI effort.

Our work is dedicated to delineating and implementing successful peer comparisons using a novel provider-specific prescribing metric – standardized antibiotic prescribing ratio (SAR) which relies on direct standardization based on patient mix indicators (M). We are also studying the relationship between changes in providers' SAR on patient outcomes. To date, the impact of hospital medicine peer comparisons on antibiotic prescribing or patient outcomes of such efforts have not been formally studied. We wish to evaluate the impact of this QI initiative and believe the results may contribute to generalizable knowledge of this process.

Preliminary data: Billing data combined with electronic antibiotic administration data and patient clinical data was used to produce a SAR for hospitalists at each of 4 EHC hospitals from January 2016 to December 2018. Direct standardization was accomplished by creating regression models to adjust for patient-level factors (age>65 years, Comorbidity Index; end stage renal disease, malignancy, pneumonia, urinary tract infection and sepsis), with data aggregated over 2-month periods; 116 hospitalists contributed to 437,303 billed patient-days. After adjustment by patient mix and entity, SAR variation remained (interquartile range of SAR 0.9-1.1) suggesting variations due to hospitalist behavior (i.e., knowledge, comfort, conformity) drive differences (K). These analyses need to be repeated during COVID-19 pandemic period to be relevant for a QI initiative in late 2022.

Study Endpoints

Primary: Change in SAR (i.e., standardized Observed: Expected ratio) (HMS provider data)

Specifically, change in median bi-monthly SAR values among all participating HMS providers between intervention and pre-intervention periods (accounting for facility effect and patient mix).

Secondary

- CDI (C. difficile infection) events among HMS patients: Likelihood (rate ratio) of CDI occurring in any HMS patient in post-intervention period is less than or equal to 1.0 (no different from baseline or protective)
- LOS (Length of stay): model attributable LOS for each HMS patient, comparing estimates of intervention period on attributable LOS.

- Readmissions: model probability of patient having readmission, and impact of intervention on readmission rates among all HMS patients, as well as stratified by providers with large reductions in SAR compared to those without large reductions in SAR.

Study Intervention/Investigational Agent

No Drug or Device is being used for this intervention.
Primary intervention.

The intervention is messaging to hospitalists via email every 2 months (bi-monthly) that contains peer comparison data of the previous 2 months and antibiotic prescribing educational content. Draft content of this message is submitted.

The detail of the content is still to be developed, but may include a relative rank in prescribing metrics, with blinding to specific Peers' identity, with department wide and facility specific goals highlighted. Those in the lowest prescribing quartile will earn the designation of "Top Performer". The usual care arm will be an educational email about simple steps to improve antibiotic prescribing sent bimonthly to all hospitalists.

Secondary Intervention (if needed).

An interim analysis will occur at the facility-level after three quarters (9 months) of intervention data to determine if high prescribers (i.e., SAR above 1.25, or 25% higher than predicted) continue to prescribe high in more than half of the intervention bimonthly prescribing periods. This would be interpreted as a lack of effect to the email-based intervention, and a secondary intervention would be offered to all high prescribers consisting of bi-monthly check in with the stewardship lead (either infectious disease pharmacist or medical director of stewardship) at each facility to provide an open forum for questions and answers about the data and any clinical practices.

Procedures Involved

Design. We will employ a quasi-experimental before-after study design, using a stepped wedge approach with 4 hospitals. Study participants will include all 147 full-time hospitalists. The intervention will be rolled out sequentially to all 4 hospitals in 3-month intervals. The intervention will last 18 months at all 4 sites.

Figure - Enrollment – facilities (EHC Entity) will be randomized to order of implementation roll out of intervention (INT), or continue baseline (BSLINE) usual care. Interim analysis (IA) would occur after 3 quarters of intervention to assess need for secondary intervention.

EHC Entity	18 months retrospective	QTR3	QTR4	QTR1	QTR2	QTR3	QTR4	QTR1	QTR2-3
First	BSLINE	INT	INT	INT	IA+INT	INT	INT	INT	INT
Second	BSLINE	BSLINE	INT	INT	INT	IA+INT	INT	INT	INT
Third	BSLINE	BSLINE	BSLINE	INT	INT	INT	IA+INT	INT	INT
Fourth	BSLINE	BSLINE	BSLINE	BSLINE	INT	INT	INT	IA+NT	INT

Analysis Plan and Outcomes.

Primary outcome is the provider-specific SAR, separately for each antibiotic grouping. Secondary outcomes include CD, readmission (< 30 days of discharge), length of stay. Changes in 2-month SAR will be estimated using an interrupted time series analysis, evaluating changes in each antibiotic grouping separately, analysis will consider hospital fixed effects. Secondary outcomes will be analyzed at the patient level among all billed encounters using logistic or negative binomial regressions. Inclusion of provider and quarter-year fixed effects will control for time-invariant provider and site characteristics and secular time trends (e.g., seasonal patient mix) and establish a difference-in-difference framework. This approach will estimate the within-provider change in patient outcomes, pre- and post-intervention. We will control for patient (e.g., sex, age, Comorbidity Index) mix, time-variant provider (e.g., census) factors.

Data Sources: The primary source of data will be the EH Clinical Data Warehouse (CDW), a repository that integrates data from multiple clinical applications within EHC and has flexibility to create customized, research-specific metrics. Outcomes for Aim 2 will also be calculated from data elements within the EH CDW (i.e., re-admissions, length of stay.)

Provider-specific SARs. Data elements will be aggregated into 2-month intervals for each of the 147 hospitalists across each hospital. Billed Days of Therapy (bDOT) and billed patient-days (bPD) derived for each antimicrobial group will be limited to the eligible dates of each patient encounter to dates billed by a hospitalist (bPD). For each period, a SAR will be determined from provider specific bDOT calculated along with predicted DOT using preliminary regression models. Regression models will be created from analyzing summary provider specific data and providers' encounter data for each 2-month period of the baseline period. Encounter summary data will be aggregate number of encounters billed by

each provider, and the % of those billed encounters in which the patient had each of the listed comorbid conditions, resulting in a summary observation without any patient PHI (i.e., data will be at the provider level, with no. and percent of encounters with each condition, and no. of DOT for all encounter in aggregate). Patient comorbidities include age, renal disease, neurologic condition, heart failure, sepsis, urinary tract infection, pneumonia, COVID-19. SAR data will be analyzed using visualization software (flexdashboard for R) to create ranking visualization and tabular reports to identify high and low risk adjusted prescribers.

Patient safety. HMS monitors re-admission rates and LOS as routine quality indicators for each provider. Monthly values will be monitored by study team on a semi-annual basis to identify potential adverse effects among HMS patients.

Data Specimen Banking

No plans for data or specimen banking for future use.

Sharing of Results with Participants

HMS providers will be receiving data on prescribing as part of the intervention, results of the impact of this QI will be shared with HMS and all providers through Departmental meetings at the tail end of the study period. There are no plans to share results with patients cared for on the HMS service.

Study Timelines

All HMS providers will be expected to participate in this QI Activity as per Department prerogative (Department chair is not a collaborator on this project, but has expressed interest in this QI and fully supportive)

- Spring 2022 - Approvals and Socialization of the QI initiative with HMS
- Spring-summer 2022 Baseline analysis and regression modeling completion
- Summer 2022 - Creation of bimonthly feedback report
- October 2022 - Roll out of intervention
- October 2023 - Data and safety monitoring, interim evaluation with HMS leadership
- March-Summer 2024 - Final analysis and internal discussions on maintaining intervention or modification
- January 2025 - Publication of results

Inclusion and Exclusion Criteria

All 147 HMS providers will be eligible to be included in the study, with summary data of all adults (≥18 years old) admitted to the HMS at either Emory University Hospital (EUH), Emory University Hospital Midtown (EUHM), Emory Johns Creek Hospital (EJCH), or Emory St. Joseph's Hospital (ESJH)

Population.

HMS providers do not overlap with any of the special populations identified by the IRB template. Moreover, the patient data summarized is agnostic to any of the identified special populations.

Vulnerable Populations

The sole purpose of this study is to evaluate impact of peer-comparison of prescribing data to HSM providers on reducing high rates of antibiotic prescribing. This study is not designed to specifically address or analyze prisoners or other vulnerable populations, and this is not a focus of the research. Our dataset will not identify if the patient is a prisoner, employee or cognitively impaired so it is possible that some of these vulnerable individuals will be included in the retrospective datasets.

Local Number of Participants

All 147 HMS providers will be eligible to be included in the study.

Recruitment Methods

The Department of Hospital Medicine will work with study team to socialize this QI initiative with all HMS providers to inform them of the process. The Department will consider this a QI initiative and all HMS providers will participate in the initiative as a QI initiative, so no recruiting materials will be utilized. However, in the 4 months leading up to the launch of the study a web-site will be launched with initiative information and a description of this study as a means to study the impact of the intervention. Two emails

will be sent by the Division Leadership with the website address and an invitation for staff to view the information. We have submitted draft contents of this website with this protocol submission.

Withdrawal of Participants

None anticipated since this is a QI initiative with oversight by HMS and the Stewardship Research Team.

Risk to Participants

There are no foreseeable risks to the HMS providers, except perhaps perceptions by peers being altered if the providers share his/her prescribing metrics and rankings.

Risk to patients cared for by the HMS providers are not foreseeable as well, as all providers will continue to practice best care and no specific treatment recommendations are coming from any aspect of this QI. Only provision of educational resources based on current EHC standards of practice

The only risk to study participants is the possibility for a breach of confidentiality via accidental disclosure of identifiable information. However, as detailed above, we will take all necessary precautions to ensure data is securely stored in a HIPAA compliant manner and only trained and approved study investigators will have access to this data.

Potential Benefits to Participants

HMS providers will likely benefit from this formal evaluation of the QI by receiving summary data on the impact the intervention had on their prescribing habits and patient outcomes.

Compensation to Participants

none

Data Management and Confidentiality

All data from this study will be stored in a secure folder on the Emory OneDrive/Teams for Business platform that is HIPAA approved and supported by the Emory Office of Information Technology. No patient-specific data will be included as part of the data set at any point, only summary counts of encounters with specific patient mix indicators and days of therapy during the encounter.

Provider specific data will be included, including name. Provider identifies are needed to remain with the bimonthly metric data to allow accurate feedback of data to the correct provider. Only approved study investigators that have completed all required CITI trainings will be allowed access to the datasets. Data will be kept for 3 years after study completion and until all manuscripts have been published and the IRB is closed out.

(Add your text)

Provisions to Monitor the Data to Ensure the Safety of Participants

There is no more than minimal risk to participants.

Provisions to Protect the Privacy Interest of Participants

Participants will have an opportunity to participate in focus groups and Departmental meetings to provide feedback to the study team, in a town hall format. The Department of Hospital Medicine will work with study team to socialize this QI initiative with all HMS providers to inform them of the process.

Economic Burden to Participants

None

Informed Consent

We are requesting a waiver of consent for this research.

Regarding HMS providers – Our research study will have no direct effect on patient care, as the QI feedback involves data from the previous 2 months with an intent to improve the providers understanding of best practice. Considering this is a QI initiative with HMS and the Antibiotic Stewardship Teams, it involves no procedures for which written consent is normally required outside of the research context.

Regarding patient data, given the retrospective nature of the study and very large number of patients that will be included in the dataset, it would be impractical, if not impossible to contact all participants for consent. We will exclude all minors (<18 years old) in the dataset. Also, all patient data remaining in the bi-monthly dataset is summarized among all encounters at the provider level, with no ability to track back any specific antibiotic use to any specific patient.

Setting

Emory University Hospital (EUH), Emory University Hospital Midtown (EUHM), Emory Johns Creek Hospital (EJCH), or Emory St. Joseph's Hospital (ESJH)

Resources Available

- Our study team includes members of the Quality Office with access to the required data and capacity to produce the bi-monthly metric. We have dedicated project management staff and study staff to implement this study as part of our CDC funded Epicenter (PEACH) funded through the cooperative agreement outlined earlier in this protocol
- The Division of Hospital Medicine Chief and Chief Quality Officer are aware of this study and supportive of this formal evaluation of the stated QI activities.

Multi-Site Research When Emory is the Lead Site

Not applicable

References

A. Magill SS, Edwards JR, Beldavs ZG, et al. Prevalence of Antimicrobial Use in US Acute Care Hospitals, May-September 2011. *JAMA*. 2014;312(14):1438–1446. doi:10.1001/jama.2014.12923

B. Wachter RM, Goldman L. 2016. Zero to 50,000 – The 20th Anniversary of the Hospitalist. N Engl J Med 375:1009-1011

C. Wiley Z, Kobaidze K, Sexton ME, Jacob JT. 2018. Hospitalists as Integral Stakeholders in Antimicrobial Stewardship. Curr Treat Options Infect Dis 10:240-248

D. Fridkin S, Baggs J, Fagan R, et al. Vital signs: improving antibiotic use among hospitalized patients. *MMWR Morb Mortal Wkly Rep*. 2014;63(9):194–200.

E. Persell SD, Friedberg MW, Meeker D, et al. Use of behavioral economics and social psychology to improve treatment of acute respiratory infections (BEARI). BMC Infect Dis. 2013;13:290.

F. Allcott H, Rogers T. The short-run and long-run effects of behavioral interventions. American Economic Review. 2012;104(10):3003-3037. doi:10.1257/aer.104.10.3003

G. Gerber JS, Prasad PA, Fiks AG, et al. Durability of benefits of an outpatient antimicrobial stewardship intervention after discontinuation of audit and Feedback.JAMA. 2014;312(23):2569-2570.

H. Meeker D, Linder JA, Fox CR, et al. Effect of behavioral interventions on inappropriate antibiotic prescribing among primary care practices.JAMA. 2016; 315(6):562-570.

I. Jung S, Sexton ME, Owens S, Spell N, Fridkin S. Variability of Antibiotic Prescribing in a Large Healthcare Network Despite Adjusting for Patient-Mix: Reconsidering Targets for Improved Prescribing. *Open Forum Infect Dis*. 2019;6(2):ofz018. Published 2019 Jan 18. doi:10.1093/ofid/ofz018

J. O'Hara LM, Masnick M, Leekha S, Jackson SS, Blanco N, Harris AD. Indirect Versus Direct Standardization Methods for Reporting Healthcare-Associated Infections: An Analysis of Central Line-Associated Bloodstream Infections in Maryland. *Infect Control Hosp Epidemiol*. 2017;38(8):989–992. doi:10.1017/ice.2017.120

K. Stevens M, Hastings SN, Markland AD, Hwang U, Hung W, Vandenberg AE, Bryan, Cross, Powers J, McGwin G, Fattouh N, Ho W, Clevenger C, Vaughan CP. Enhancing

Quality of Provider Practices for Older Adults in the Emergency Department (EQUiPPED). J Am Geriatr Soc. 2017 Jul;65(7):1609-1614. doi: 10.1111/jgs.14890.

L. Szymczak JE. 2018. Are Surgeons Different? The Case for Bespoke Antimicrobial Stewardship. Clin Infect Dis; doi: 10.1093/cid/ciy847

M. Charani E, Tarrant C, Moorthy K, Sevdalis N, Brennan L, Holmes AH. 2017. Understanding antibiotic decision making in surgery—a qualitative analysis. Clin Microbiol Infect 23:752–60.

N. James Baggs, John A Jernigan, Alison Laufer Halpin, Lauren Epstein, Kelly M Hatfield, L Clifford McDonald, Risk of Subsequent Sepsis Within 90 Days After a Hospital Stay by Type of Antibiotic Exposure, *Clinical Infectious Diseases*, Volume 66, Issue 7, 1 April 2018, Pages 1004–1012, https://doi.org/10.1093/cid/cix947

O. Morris, A., Calderwood, M., Fridkin, S., Livorsi, D., McGregor, J., Mody, L., . . . Tamma, P. (2019). Research needs in antibiotic stewardship. *Infection Control & Hospital Epidemiology, 40*(12), 1334-1343. doi:10.1017/ice.2019.276

Impact of peer-comparison feedback on hospitalists' antibiotic prescribing of broad-spectrum antibiotics: a stepped wedge randomized clinical trial in an academic healthcare network

Statistical Analysis Plan

Contents

1. *Study Objective* 18
2. *Intervention* 18
3. *Study Design* 19
4. *Study Population and Sample Size* 20
5. *Study Measures and Outcomes* 20
6. *Safety Monitoring* 21
7. *Statistical Analysis* 21

1. Study Objective

Inappropriate antibiotic prescribing in both primary care and inpatient settings contributes to antibiotic resistance and associated adverse outcomes like *Clostridioides difficile* infection (CDI) and increased healthcare costs.(C D C 2019; Jit et al. 2020). While antibiotic stewardship interventions, such as provider education and feedback, have shown promise in ambulatory settings, their translation to inpatient care faces unique challenges. **In this study, we aim to determine the impact of using an automated peer comparison report of antibiotic prescribing on hospitalists prescribing rates without negative impact on patient outcomes** in an academic network of hospitals in Atlanta, Georgia.

2. Intervention

The intervention will comprise two components: (1) a one-time educational session on antibiotic de-escalation and (2) bimonthly email feedback reports. Educational sessions, led by facility stewardship leads, will focus on evidence-based recommendations for presumed pneumonia and urosepsis, including indications for empiric anti-pseudomonal coverage and use of the hospital's antibiotic prescribing assistance tool. Feedback reports will reinforce these indications and will provide a link to the tool.

Provider-specific feedback reports will use observed-to-expected ratios (OERs), derived in part from EPIC Clarity System clinical and billing data. Generalized linear models will be used to generate OERs, adjusted for the proportions of patients with sepsis, end-stage renal disease, and urinary tract infections. These risk adjustments will be based on a prior study identifying factors consistently associated with broad-spectrum hospital-onset (BS-HO) antibiotic prescribing (Onwubiko et al. 2024).

Bimonthly, the stewardship research team will automatically email private PDF reports to each provider. Report content and formatting will be informed by CP-FIT's key feedback elements and will be assessed through cognitive interviews with 13 providers conducted during the development phase, with corrective changes to be implemented based on structured interview questions.

One week after the third prescribing report is sequentially sent (six months after intervention initiation), the antibiotic stewardship team will email a single-question Microsoft Form survey to providers, seeking confirmation of whether they viewed the previous reports. Non-responders will receive email reminders from the hospital medicine faculty liaison, who will then personally follow up with remaining non-responders to encourage completion.

3. Study Design

We plan to use a Randomized Step Wedge Cluster Design (R-SWCD) to evaluate the effect of the peer comparison feedback report on antibiotic prescribing (BS-HO group). The study will be conducted in 4 hospitals (clusters) in Atlanta, Georgia. Of note, pending discussion with Hospital Medicine Leadership, the intervention may also be rolled out at an additional hospital, although lack of retrospective data would not allow inclusion of this facility in the full analysis. Hospitals will begin in the control condition and end in the intervention condition, wherein, all hospitals will receive the intervention and cross over to the intervention arm in a random order (Figure 1). Randomization will occur at the hospital level rather than at the provider level to avoid contamination and spillover effects. Providers within a hospital are not fully independent of each other, groups tend to form because of certain selection factors, and so providers tend to be more similar to each other with respect to potential confounders.

Outcomes linked to the same provider will be assessed repeatedly on a bi-monthly bases in each hospital over a two-year time period (2023-2024). Hospitals will cross-over to the intervention arm sequentially, resulting in 4 steps with an equal step length of 2 months. Following a baseline period (January 1 - June 30, 2023), the first cluster will begin the intervention on July 1, 2023. The final cluster will transition by March 1, 2024, with intervention continuing through December 31, 2024 in all hospitals.

A step wedge design, which involves the randomized and sequential roll out of the intervention to hospitals over time was chosen as it can model and adjust for any underlying temporal trends and results in all hospitals receiving the intervention **which is desirable from a healthcare quality improvement and antimicrobial stewardship perspective.** Randomization of the order of implementation reduces confounding from baseline cluster-level factors. Lastly, gradual roll out of the intervention will allow us to identify and address any implementation bottlenecks.

Figure 1. Stepped-Wedge interventional study design. Abbreviations: E, educational session; PF, Provider Feedback. Grey boxes reflect intervention periods with bi-monthly PF reports.

	2023				2024	
	Jan-Jun 23	Jul-23	Sep-23	Nov-23	Jan-24	May-24	Jul to Dec 24
Hospital A		E + PF	PF	PF	PF	PF	PF
Hospital B			E + PF	PF	PF	PF	PF
Hospital C				E + PF	PF	PF	PF
Hospital D					E + PF	PF	PF
Hospital E						E + PF	PF

4. Study Population and Sample Size

The study will focus on hospitalists as the primary antibiotic prescriber. The list of hospitalists will be identified and fixed at study onset and updated semi-annually. Updates from Hospital Medicine Service Administration will be obtained on any staffing changes, although staff turnover is not expected to exceed 10%. A secondary analysis may be performed on new staff who join during the study implementation phase. Hospitalists typically do not practice in more than one hospital and so we do not expect any contamination across hospitals. The study will exclude nocturnists (night-shift hospitalists) as they have less control over antibiotic prescribing (n = 11; 8% of all hospitalists). The final study sample will include a total of 125 hospitalists (15-40 hospitalists per hospital) across 4 Emory hospitals.

5. Study Measures and Outcomes

Data from January 2023 to December 2024 will be extracted for inpatients at all hospitals with Hospital Medicine Services. For each hospitalist, billing data will be used to identify patient encounters and to calculate billed patient-days (bPD). Antibiotic prescribing data from electronic medication administration records (eMAR) will capture days of antibiotic therapy (DOT) for NHSN-defined broad-spectrum hospital-onset (BS-HO) agents per bPD.

Patient characteristics linked to each encounter will include age, microbiology results (e.g., bacteremia), ICD-10–based antibiotic indications (e.g., pneumonia, COVID-19, sepsis, urinary tract infection), and comorbidities, enabling calculation of Elixhauser comorbidity indices. Generalized linear models will be used to generate provider-level observed-to-expected ratios (OERs). In each model, bPD will remain continuous, while other predictors will be categorized by quintiles based on their relationship with DOT. Based on input from Hospital Medicine Services leadership and focus groups, hospital-specific models will be used to calculate OERs in order to maximize provider credibility of the metric.

The primary outcome will be the observed provider-specific antibiotic prescribing rate, defined as billed DOT of BS-HO antibiotics per 1,000 billed patient-days. This measure alone will not be risk adjusted; however, patient- and provider-level characteristics will be adjusted for and included as covariates in the regression model described below. For illustrative (descriptive) purposes, risk-adjusted provider-specific OERs will also be estimated, with point estimates (means) and variance plotted over time by facility. OERs will not be modeled directly as regression outcomes.

6. *Safety Monitoring*

Secondary outcomes and safety metrics will be assessed at the patient-encounter level and will include Clostridioides difficile infection occurring during the inpatient stay or within 8 weeks after discharge, 30-day readmission for any reason, in-hospital mortality, and prolonged length of the index hospitalization (defined as >7 days).

To maximize detection of potential safety signals, secondary outcome analyses will be restricted to patients receiving hospital medicine care who are discharged with an ICD-10 code indicating community-acquired pneumonia, urinary tract infection, or urosepsis—the two primary treatment indications targeted by the intervention. During interim analyses, all in-hospital deaths will be reviewed by the study team to assess any potential association with antibiotic de-escalation.

7. *Statistical Analysis*

For the primary outcome analysis, a generalized linear mixed-effects model with a negative binomial distribution and log link will be used to evaluate the effect of the intervention on antibiotic prescribing rates over time. The observed DOT will be modeled as a function of provider receipt of feedback reports, time (bimonthly period) to account for secular trends and time-varying confounding, and a random facility effect to account for clustering of providers within hospitals. A random intercept for each provider nested within facility will be included to account for correlation among repeated observations and the hierarchical data structure. The intra-cluster correlation coefficient (ICC) will be estimated to quantify the proportion of total variance attributable to between-cluster differences. Models will adjust for patient-mix characteristics as covariates.

Sensitivity analyses of the primary outcome will be conducted among the subset of providers who indicate that they received and reviewed the feedback reports, to account for potential implementation bias.

Secondary outcomes will be analyzed at the patient level among all patients receiving care from hospital medicine services and discharged with ICD-10 codes for pneumonia or urinary tract infection, as this group is expected to be most vulnerable to changes in antibiotic de-escalation practices. Safety outcomes will be analyzed using generalized mixed-effects logistic regression models to evaluate intervention effects over time, accounting for repeated hospitalizations within patients and clustering by facility. Covariates will include time (to account for seasonality) and patient-level indicators (COVID-19, influenza, Elixhauser comorbidity index, and sepsis). All analyses will be performed using R Statistical Software (version 4.2.0; R Core Team, 2021).
