# Supplementary material for: Association Between Peer Comparison Feedback and Hospitalist Antibiotic Prescribing
Source: JAMA Netw Open. 2026 Apr 28;9(4):e269504. doi: 10.1001/jamanetworkopen.2026.9504 (PMC13126217; doi:10.1001/jamanetworkopen.2026.9504)
Supplement: Supplement 3. — Data Sharing Statement [file jamanetwopen-e269504-s003.pdf]

# Data Sharing Statement

Witt. Association Between Peer Comparison Feedback and Hospitalist Antibiotic Prescribing. *JAMA Netw Open*. Published April 28, 2026. doi:10.1001/jamanetworkopen.2026.9504

**Data**
**Data available:** Yes
**Data types:** Deidentified participant data
**How to access data:** The datasets generated and/or analyzed for the current study are to be available in the Emory Dataverse repository (URL: https://dataverse.unc.edu/dataverse/Emory). To eliminate risk of identification, hospital facility characteristics (bed size, location, etc.) will be suppressed to minimize identification of institution. All personally identifying information (e.g., names, addresses, phone numbers, etc.) are absent from the data in compliance with HHS Guidance Regarding Methods for De-identification of Protected Health Information in Accordance with the HIPAA Privacy Rule.
**When available:** With publication

**Supporting Documents**
**Document types:** None

**Additional Information**
**Who can access the data:** Researchers whose proposed use of the data has been approved)
**Types of analyses:** For a specified purpose
**Mechanisms of data availability:** After approval of a proposal
